# Primary cutaneous blastomycosis after eyebrow wax burn

**DOI:** 10.1016/j.jdcr.2022.02.019

**Published:** 2022-03-02

**Authors:** Sarah A. Ibrahim, Megha Trivedi, David C. Reid

**Affiliations:** a Rush Medical College, Chicago, Illinois; b Division of Dermatology, Rush University Medical Center, Chicago, Illinois

**Keywords:** *Blastomyces dermatitidis*, blastomycosis, case report, cutaneous blastomycosis, diagnosis

## Introduction

Blastomycosis is one of three major thermally dimorphic mycoses endemic to North America, particularly so in the Mississippi and Ohio River valleys, as well as in the Great Lakes area.^1^, ^2^, ^3^ The organism responsible for the disease, *Blastomycosis dermatitidis*, lives as a benign mold in the environment and transforms to a pathologic yeast after inoculation. Infection is typically acquired from inhalation of the organism but can also occur from direct inoculation after trauma.^1^ Those who engage in work outdoors are most likely to be infected.^1^

Blastomycosis has a predilection for the lung, and the pathophysiology typically involves a primary pulmonary infection. Cutaneous manifestations of pulmonary blastomycosis are common and are reported in up to 60% of patients.^1^ However, primary cutaneous blastomycosis is much less frequently reported.

We present the case of primary cutaneous blastomycosis in a middle-aged woman in the Midwest US, who had waxed her eyebrows one week prior to lesion development. Notably, the patient had developed a wax burn following the treatment, a known risk of eyebrow waxing, with resultant hypopigmentation in the treated area. The diagnosis of blastomycosis was made after a high clinical index of suspicion and confirmed after twenty six days with positive tissue culture.

## Case report

**Fig 1:**
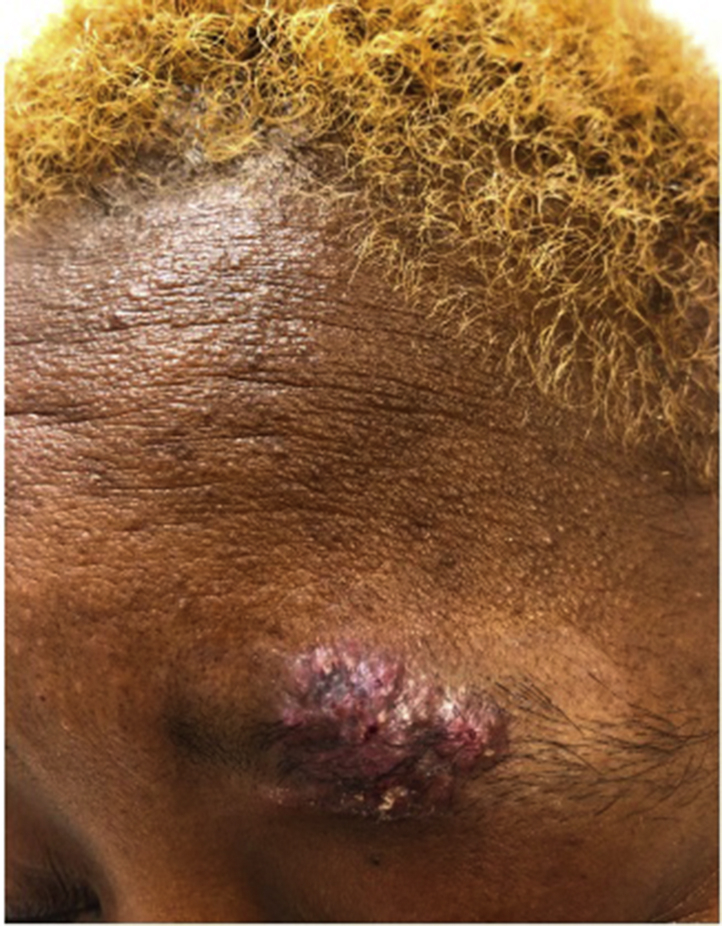
Blastomycosis. Physical examination of the lesion revealed a well-demarcated, violaceous, verrucous plaque with raised borders and an exudative crust at the base.

**Fig 2:**
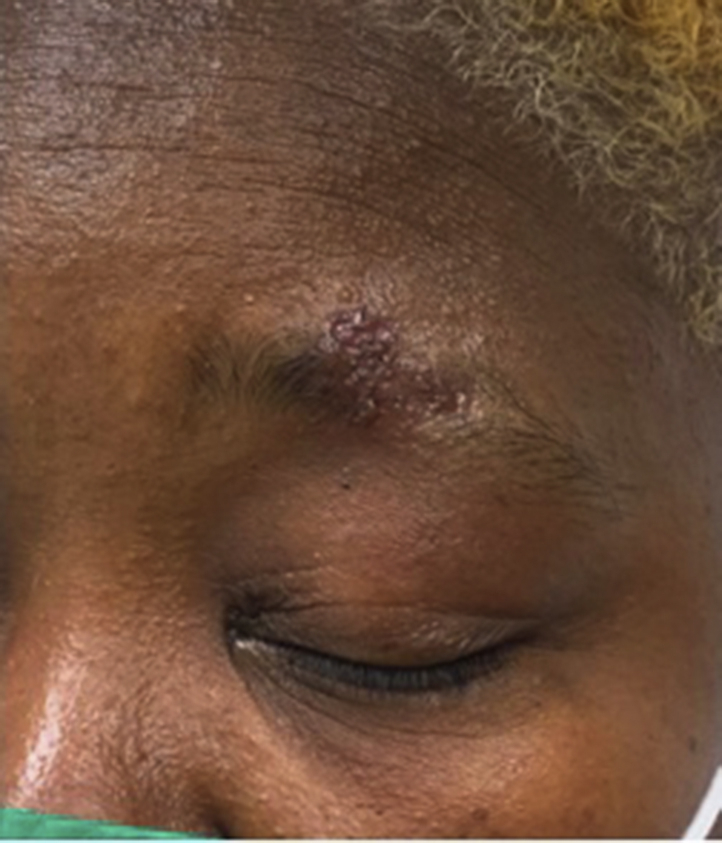
Same lesion as in Fig 1 above the left eyebrow after 33 days of treatment with fluconazole.

A 50-year-old African American female with no contributing medical history presented with a 1-month history of a lesion over her left eyebrow. Prior to the development of the lesion, she reported fatigue for several days, but otherwise had no fevers, chills, weight loss, night sweats, or shortness of breath. One week prior to lesion onset, she had waxed her eyebrows, with pruritus and pain in the area subsequent to the waxing treatment. She denied any other trauma or treatments to the area. She worked as a janitor at an academic facility and denied engaging in recent outdoor activities. Physical examination demonstrated a well-demarcated, violaceous, verrucous plaque with raised borders and an exudative crust at the base (Figs 1 and 2). Surrounding the plaque, in the area of the wax treatment, there was a well-defined hypopigmented patch, consistent with postinflammatory hypopigmentation.

The lesion morphology and development following trauma prompted a high clinical suspicion for a deep fungal infection, such as blastomycosis or cutaneous tuberculosis. Other differential diagnoses included Majocchi granuloma and inflammatory dermatoses, including sarcoidosis.

**Fig 3:**
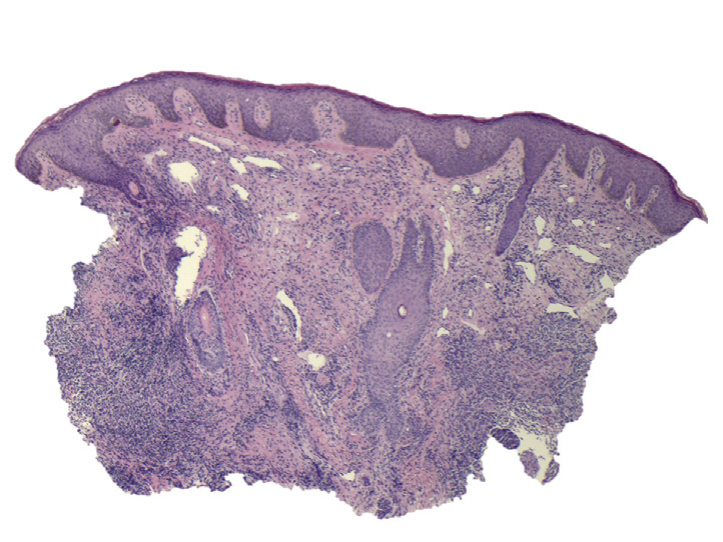
Punch biopsy demonstrating pseudoepitheliomatous hyperplasia in the epidermal layer (hematoxylin-eosin stain; original magnification ×4).

A punch biopsy of the lesion demonstrated nonspecific inflammatory infiltrates around hair follicles and pseudoepitheliomatous hyperplasia in the epidermal layer (Fig 3). Periodic acid–Schiff, Gomori methenamine silver, and acid-fast stains were negative. Ten days after biopsy, tissue cultures had not grown any organisms.

Despite negative special stains, a high clinical suspicion for a deep fungal infection remained. The patient was empirically started on fluconazole, and a small decrease in lesion size was observed. Twenty six days after biopsy, fungal cultures grew *Blastomyces dermatitidis*. A diagnosis of primary cutaneous blastomycosis was made, and treatment was begun with itraconazole. The patient was referred to infectious diseases. A systemic workup was done, including a lung examination and computed tomography of the brain and cervical spine, which were negative.

## Discussion

Primary cutaneous blastomycosis is rare. Although the infectivity of primary cutaneous blastomycosis has not been reported, fewer than 50 cases have been documented.^3^, ^4^, ^5^ Much more frequently, cutaneous manifestations of primary pulmonary disease are reported. Blastomycosis occurs in both immunocompetent and immunosuppressed patients. Because of the potential for more serious infections in immunocompromised hosts, patient investigation should include review for HIV/AIDS, history of organ transplantation, hematologic malignancy, diabetes, and long-term glucocorticoid usage. In the presented case, the patient’s negative systemic workup strongly suggested a primary infection rather than disseminated disease after pulmonary inoculation. Supporting this diagnosis were her history of recent eyebrow waxing and postinflammatory hypopigmentation, suggesting possible micro-trauma to the skin, which would have allowed for direct inoculation with *Blastomyces* conidia.

Cases of primary cutaneous blastomycosis have typically been documented after exposure to soil-covered items or animal-related products. For example, reported cases have included injuries from tree bark and inoculation from a woodworking blade.^4^^,^^5^ However, this is the first documented case of primary cutaneous blastomycosis arising after waxing-induced trauma.

Depilatory waxing, particularly wax burns, has been shown to significantly reduce the thickness of the stratum corneum, resulting in a disrupted epidermis and rendering the skin susceptible to contact irritants and infection.^6^^,^^7^ Waxing-induced trauma is more frequently documented in women and is associated with inappropriate reheating of wax.^8^ Notably, wax products contain rosins, or by-products derived from plants, which have not yet been associated with blastomycosis but may possibly be an infectious source.^9^

Because of the varying morphologies and often absent pulmonary symptoms, diagnosis of primary cutaneous blastomycosis is challenging. In the presented case, the lesion characteristics did raise concern for a deep fungal infection and aided in driving diagnostic workup. Lesions are classically verruciform, with raised, irregular borders, and central crust or ulceration.^3^ Histologically, blastomycosis can be diagnosed by identification of intracellularly or extracellularly located oval, multinucleated yeast cells with a diameter of 8 to 15 μm and with broad-based budding.^2^ While histology can aid diagnosis, culture is the most sensitive diagnostic tool and is the gold standard for definitive diagnosis of blastomycosis.^2^ Growth of *B. dermatitidis* is usually seen after 5-10 days but may not appear until 30 days.^2^ In the presented case, culture of the lesion was the most important diagnostic tool for diagnosing blastomycosis, despite the fact that it took 4 weeks to appreciate growth. Additionally, antigen testing via urine specimen collection can be used for diagnosis. However, while the sensitivity is high (92.9%), the specificity is low, and this method is less reliable than culture.^2^

Treatment of blastomycosis varies depending on the severity of extrapulmonary disease. For patients affected by mild-to-moderate disease, a 3-day course of oral itraconazole 200 mg three times daily is recommended, followed by 1-2 times daily for 6-12 months.^10^

## Conflicts of interest

None disclosed.
